# The role of renal dipeptidyl peptidase-4 in kidney disease: renal effects of dipeptidyl peptidase-4 inhibitors with a focus on linagliptin

**DOI:** 10.1042/CS20180031

**Published:** 2018-02-28

**Authors:** Keizo Kanasaki

**Affiliations:** 1 Department of Diabetology and Endocrinology, Kanazawa Medical University, Uchinada, Japan; 2 Division of Anticipatory Molecular Food Science and Technology, Medical Research Institute, Kanazawa Medical University, Uchinada, Japan

**Keywords:** chronic kidney disease, dipeptidyl peptidase- 4 inhibitors, type 2 diabetes

## Abstract

Emerging evidence suggests that dipeptidyl peptidase-4 (DPP-4) inhibitors used to treat type 2 diabetes may have nephroprotective effects beyond the reduced renal risk conferred by glycemic control. DPP-4 is a ubiquitous protein with exopeptidase activity that exists in cell membrane-bound and soluble forms. The kidneys contain the highest levels of DPP-4, which is increased in diabetic nephropathy. DPP-4 inhibitors are a chemically heterogeneous class of drugs with important pharmacological differences. Of the globally marketed DPP-4 inhibitors, linagliptin is of particular interest for diabetic nephropathy as it is the only compound that is not predominantly excreted in the urine. Linagliptin is also the most potent DPP-4 inhibitor, has the highest affinity for this protein, and has the largest volume of distribution; these properties allow linagliptin to penetrate kidney tissue and tightly bind resident DPP-4. In animal models of kidney disease, linagliptin elicited multiple renoprotective effects, including reducing albuminuria, glomerulosclerosis, and tubulointerstitial fibrosis, independent of changes in glucagon-like peptide-1 (GLP-1) and glucose levels. At the molecular level, linagliptin prevented the pro-fibrotic endothelial-to-mesenchymal transition by disrupting the interaction between membrane-bound DPP-4 and integrin β1 that enhances signaling by transforming growth factor-β1 and vascular endothelial growth factor receptor-1. Linagliptin also increased stromal cell derived factor-1 levels, ameliorated endothelial dysfunction, and displayed unique antioxidant effects. Although the nephroprotective effects of linagliptin are yet to be translated to the clinical setting, the ongoing Cardiovascular and Renal Microvascular Outcome Study with Linagliptin in Patients with Type 2 Diabetes Mellitus (CARMELINA®) study will definitively assess the renal effects of this DPP-4 inhibitor. CARMELINA® is the only clinical trial of a DPP-4 inhibitor powered to evaluate kidney outcomes.

## Introduction

The global burden of diabetes is escalating at an alarming rate, with an estimated 425 million people worldwide afflicted with the disease in 2017 [1], mostly (90−95%) with type 2 diabetes [2]. Furthermore, global diabetes prevalence is forecast to increase to 629 million people by 2045 [1]. Microvascular complications resulting from hyperglycemia, including kidney disease, are major clinical sequelae of type 2 diabetes. Consequently, approximately 50% of people with type 2 diabetes also have chronic kidney disease [3], making diabetes the leading cause of chronic kidney disease [4].

Intensive glycemic control has been shown to reduce the risk of kidney disease and other microvascular complications of type 2 diabetes in large clinical outcomes studies such as the UK Prospective Diabetes Study (UKPDS), the Action to Control Cardiovascular Risk in Diabetes (ACCORD) study, and the Action in Diabetes and Vascular Disease: Preterax and Diamicron Modified Release Controlled Evaluation (ADVANCE) study [5–10]. However, the residual risk of kidney complications remains substantial even with current standard of care, including tight glycemic control, and people with diabetes are three times more likely to die from kidney disease than those without diabetes [11]. No new treatments for diabetic nephropathy have emerged since the angiotensin-receptor blockers irbesartan and losartan demonstrated efficacy in this indication in 2001 [12,13]. Since then, several novel pharmacotherapeutic approaches for diabetic kidney disease have failed to exhibit beneficial effects in carefully controlled clinical trials [14]. As a consequence, there is a greater interest in potential renoprotective effects of type 2 diabetes drugs that are independent of their glucose-lowering properties – so-called pleiotropic effects. Recently, members of the newest class of oral glucose-lowering drugs – sodium-glucose cotransporter-2 (SGLT2) inhibitors – appeared to slow the progression of diabetic kidney disease in exploratory analyses of cardiovascular outcomes studies [15,16]. If these renoprotective effects are confirmed in clinical trials designed primarily to evaluate renal outcomes, SGLT2 inhibitors may become the standard of care for diabetic nephropathy together with angiotensin-converting enzyme inhibitors and angiotensin-receptor blockers.

Dipeptidyl peptidase-4 (DPP-4) inhibitors are also one of the newer types of oral glucose-lowering drug licensed for type 2 diabetes, with the first-in-class sitagliptin having been approved in 2006 in the United States. DPP-4 inhibitors, also known as gliptins, are orally administered medications that have moderate glycemic efficacy and carry low risk for hypoglycemia or weight gain [17]. Emerging evidence suggests that DPP-4 inhibitors may also have renoprotective effects that are independent of their glucose-lowering properties [18,19]. Despite their common mechanism of action, DPP-4 inhibitors are a chemically diverse class of molecules, whose contrasting structures result in clinically important differences in pharmacology [20].

Amongst the globally marketed DPP-4 inhibitors, linagliptin is of particular interest with respect to pleiotropic renoprotective effects as it is the only such drug to be excreted predominantly by non-renal pathways [21]; hence it does not require dose adjustment for chronic kidney disease (or any other intrinsic or extrinsic factor) [20,22,23]. In contrast, sitagliptin, saxagliptin, alogliptin, and vildagliptin are excreted mainly by the kidneys [20], which necessitates their dose adjustment in renally impaired patients as a safety precaution [24–27]. Furthermore, linagliptin is the first and so far only DPP-4 inhibitor to be evaluated in a randomized clinical trial designed to robustly assess renal outcomes: the ongoing Cardiovascular and Renal Microvascular Outcome Study with Linagliptin in Patients with Type 2 Diabetes Mellitus (CARMELINA®; 20ClinicalTrialsgov: NCT01897532).

This review describes the accumulating body of evidence suggesting that linagliptin may have protective effects against diabetic nephropathy, with a focus on preclinical studies and putative molecular mechanisms.

## More than just an enzyme: the diverse biology of DPP-4

**Figure 1:**
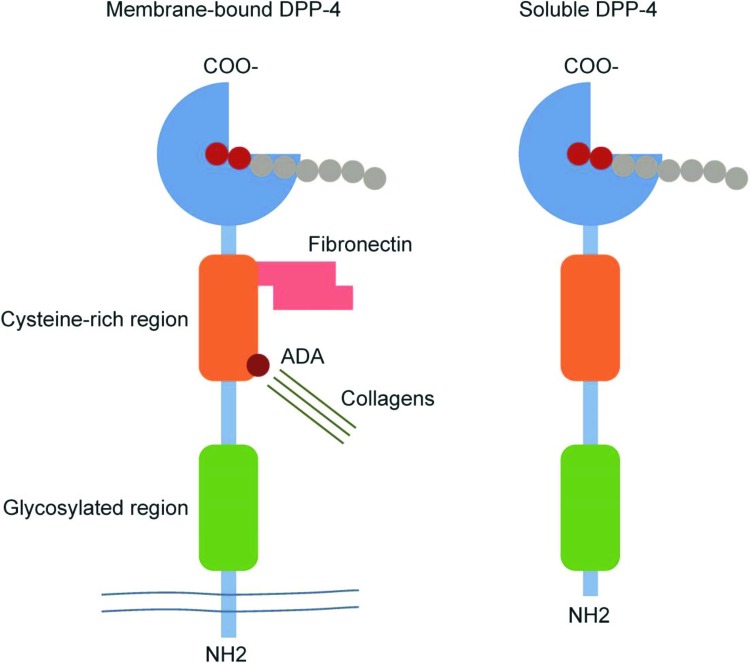
Membrane-bound DPP-4 and soluble DPP-4 Catalytically active DPP-4 is liberated from the plasma membrane to produce a soluble circulating form that lacks the intracellular tail and transmembrane regions and accounts for a substantial proportion of DPP-4 activity. In addition to its exopeptidase activity, DPP-4 also functions as a binding protein which can bind with fibronectin and adenosine deaminase (ADA), amongst other protein-binding partners. Republished with permission from Shi et al. (2016) [28], permission conveyed through Copyright Clearance Center, Inc.

DPP-4, also known as CD26 and glycoprotein gp110, is a 110-kDa glycoprotein with serine exopeptidase activity (Enzyme Commission number 3.4.14.5) [28]. The catalytic activity of DPP-4 removes the N-terminal dipeptide from peptides containing proline or alanine in the second position [28]. Structurally, monomeric DPP-4 is a type II transmembrane protein of 766 amino acid residues that consists of a short intracellular N-terminal tail, a hydrophobic transmembrane segment, and an extracellular portion accounting for the bulk of the protein; the extracellular portion contains a glycosylated region, a cysteine-rich region, and the catalytic domain at the C-terminus (Figure 1) [28].

In addition to the transmembrane form, DPP-4 also exists as a slightly smaller, soluble form (727 residues) that lacks the intracellular and transmembrane regions of the membrane-bound form but retains the extracellular portion, including the catalytic domain [28]. Membrane-bound DPP-4 exists mainly as a dimer although tetramers of two membrane-bound forms and two soluble forms can also form [29,30]. Membrane-bound DPP-4 is a ubiquitous protein that is present in most organs in a range of different tissues and cell types, including immune cells and vascular endothelial cells [28,31]. Soluble DPP-4 is found in the blood and most other body fluids [28,31], and is thought to arise from shedding of the membrane form, although its precise source has not been delineated [28,32,33].

DPP-4 was discovered over 50 years ago [34], but its complex biology is still being unraveled. It is a multifunctional protein that was first characterized as a T-cell differentiation antigen (CD26) [28]. Distinct from its enzymatic activity, DPP-4 is involved in multiple protein–protein interactions, including association with adenosine deaminase in most tissues and with the HIV envelope glycoprotein gp120 on T lymphocytes [28]. Its other known protein–protein interaction partners include the C-X-C chemokine receptor type 4 (CXCR4) chemokine receptor, the CD45 tyrosine phosphatase, the sodium-hydrogen exchanger-3 (NHE3), fibronectin, collagen, caveolin-1, and the mannose-6-phosphate/insulin-like growth factor II receptor [28,31,35]. Through these physical interactions, DPP-4 is involved in diverse biological processes such as immune system modulation (including T-cell costimulation), activation of intracellular signal transduction pathways, natriuresis, cell–cell interactions, cellular interactions with the extracellular matrix, and cellular entry of viruses such as HIV and the Middle Eastern respiratory syndrome coronavirus [28,36].

The enzymatic activity of DPP-4 has been shown to cleave a wide variety of biopeptides in *in vitro* assays, but few of these have been established as *bona fide* physiological substrates [32]. The most well-known of the latter are the peptide hormones glucagon-like peptide (GLP)-1 and glucose-dependent insulinotropic peptide, which are responsible for the incretin effect, i.e. the amplification of insulin secretion following oral but not intravenous glucose despite similar levels of blood glucose. It is thought that DPP-4 inhibitors elicit their antihyperglycemic effects predominantly by preventing degradation of GLP-1), although other mechanisms may also be involved [37]. Other confirmed physiological substrates of DPP-4 include stromal cell-derived factor (SDF)-1, GLP-2, peptide tyrosine–tyrosine (PYY), and substance P [32].

## DPP-4 in kidney disease: active agent or benign bystander?

Although DPP-4 is present throughout most of the body, its levels vary widely between different organs and tissues [28,31,38]. Amongst the organs, the highest amounts of DPP-4 activity per gram of tissue are found in the kidneys [33,38,39]. Levels of both membrane-bound DPP-4 protein and plasma DPP-4 enzymatic activity are altered in several pathophysiological states, including cancer, inflammation, infections, immune disorders, type 2 diabetes, and kidney disease [28,31]. The increased levels of soluble DPP-4 in type 2 diabetes seem to be at least partly derived from proteolytic processing of membrane-bound DPP-4 by kallikrein-related peptidase 5 on circulating CD4^+^ T helper (Th)17 cells [40].

In the kidneys, DPP-4 expression and enzymatic activity has been demonstrated to occur in several tissue types in healthy organs and under disease conditions. In rats, DPP-4 was reported to be present in glomerular podocytes and proximal tubules of the kidneys [39,41]. Notably, membrane-bound DPP-4 forms a complex with NHE3 in the brush border of renal proximal tubules where it appears to modulate NHE3-mediated Na^+^/H^+^ exchange to reduce natriuresis [42,43]. Separately, the exopeptidase activity of proximal tubule-located DPP-4 enables reabsorption of proline-containing oligopeptides [44]. *DPP-4* mRNA, protein, and enzymatic activity were detected in preglomerular microvascular smooth muscle cells and glomerular mesangial cells from spontaneously hypertensive and normotensive rats [45]. In rats either fed high-fat diets or treated with streptozotocin (STZ) to induce diabetes, DPP-4 was overexpressed in renal tubular cells [46]. Interestingly, DPP-4 deficiency protected rat kidneys from acute ischemia–reperfusion injury [47].

In humans, DPP-4 expression and enzymatic activity was found to be present in the glomerulus (primarily in podocytes) only under pathological renal conditions and not in healthy kidneys [48–50]. Interferon-γ, an inflammatory cytokine, elicited expression of DPP-4 in human glomerular epithelial cells [51]. Furthermore, exposure of human glomerular endothelial cells to high glucose concentrations *in vitro* increased *DPP-4* mRNA and enzymatic activity [52]. In contrast, DPP-4 was found to be present on the luminal side of the brush border membrane of proximal tubular cells in healthy human kidneys [53,54]. Interestingly, urinary DPP-4 activity was found to be significantly higher in individuals with type 2 diabetes and albuminuria compared with non-albuminuric diabetes patients or healthy individuals [55,56]. Furthermore, a regression analysis of relationships between stages of chronic kidney disease and serum levels of 10 proteases found that only angiotensin-converting enzyme 2 and DPP-4 activities significantly correlated with estimated glomerular filtration rate (eGFR); in both cases, the relationship was inverse such that patients with the highest DPP-4 and angiotensin-converting enzyme 2 activities exhibited the lowest eGFR [57]. Other studies have also found a correlation between increased DPP-4 activity and diabetic and non-diabetic kidney disease [58–60].

Based on these studies, it seems plausible that DPP-4 plays a pathological role in diabetic nephropathy, although reverse causation cannot be ruled out. Consequently, there is much interest in the effects of DPP-4 inhibitors such as linagliptin in this disease.

## Effects of linagliptin in animal models of kidney disease

Linagliptin has been extensively investigated in animal models of diabetic nephropathy, as well as non-diabetic kidney disease (Table 1). In a mouse model of hypertensive diabetes, linagliptin monotherapy reduced glomerulosclerosis and renal oxidative stress, while the combination of linagliptin and the angiotensin-receptor blocker telmisartan reduced albuminuria more than telmisartan alone [61]. In a rat model of type 1 diabetes in which serum DPP-4 levels were elevated, linagliptin reduced levels of advanced glycation end products (AGEs) and their receptor (RAGE), as well as reducing albuminuria, lymphocyte infiltration into glomeruli, and 8-hydroxy-2′-deoxyguanosine levels in the kidney, a marker of renal oxidative stress. These changes occurred without alterations in blood glucose levels [62]. A follow-up study found that DPP-4 deficiency mimicked these effects, suggesting that DPP-4 inhibition itself was responsible for reducing AGE-RAGE signaling, rather than any off-target effects of linagliptin [63]. Similarly, in a mouse model of type 2 diabetes, linagliptin reduced albuminuria and kidney damage without affecting blood glucose levels [50]. Notably, in a study conducted by the author and colleagues, linagliptin inhibited tubulointerstitial fibrosis in a mouse model of diabetic nephropathy characterized by extensive fibrosis (STZ-induced diabetic CD-1 mice), as well as reducing glomerulosclerosis and albuminuria [64]. This study and its follow-on investigation [65] have shed light on molecular mechanisms for the renoprotective effects of linagliptin, and this research will be discussed further below. Intriguingly, a separate study found that linagliptin lowered albuminuria in diabetic mice with GLP-1 receptors (*Glp1r^+/+^*) but not in those lacking the GLP-1 receptor (*Glp1r^−/−^*) [66]. However, linagliptin treatment normalized kidney pathology, reduced renal oxidative stress, increased natriuresis, and up-regulated expression of SDF-1 in both *Glp1r^+/+^* and *Glp1r^−/−^* mice [66]. In a rat model of early diabetic nephropathy (STZ-induced diabetic Sprague–Dawley rats), treatment with linagliptin reduced albuminuria without affecting blood glucose levels [67]. Linagliptin also attenuated expression of vascular endothelial growth factor (VEGF) and the oxidative stress markers NADPH oxidase (NOX) 2 (NOX2) and NOX4 [67].

**Table 1:** Renoprotective effects of linagliptin in animal models of diabetic nephropathy and non-diabetic kidney disease

Animal model	Effects of linagliptin	Reference
**Diabetic**		
STZ-diabetic, eNOS knockout C57BL/6J mouse	↓Glomerulosclerosis (as monotherapy)	Alter et al. (2012) [61]
	↓Renal oxidative stress (as monotherapy)	
	↓Albuminuria (combined with telmisartan)	
STZ-diabetic Sprague–Dawley rat	↓Albuminuria	Nakashima et al. (2014) [62]
	↓AGE, RAGE in the kidney	
	↓Renal oxidative stress	
	↓Lymphocyte infiltration of glomerulus	
*db/db* C57BL6 mouse	↓Albuminuria	Sharkovska et al. (2014) [50]
	↓Glomerulosclerosis	
	↓Tubulointerstitial fibrosis	
	↓Podocyte damage	
STZ-diabetic CD-1 mouse	↓Glomerulosclerosis	Kanasaki et al. (2014) [64]
	↓Tubulointerstitial fibrosis	
	↓Albuminuria	
*Glp1r^+/+^* and *Glp1r^−/−^* diabetes-prone Akita mice	↓Albuminuria (only in *Glp1r^+/+^* mice)	Takashima et al. (2016) [66]
	↓Glomerulosclerosis	
	↓Periglomerular fibrosis	
	↓Podocyte loss	
	↓Renal oxidative stress	
STZ-diabetic Sprague–Dawley rat	↓Albuminuria	Gill et al. (2017) [67]
	↓Renal oxidative stress	
**Non-diabetic**		
Wistar rats with 2-kidney-1-clip surgery	↓Oxidative stress	Chaykovska et al. (2013) [68]
Zucker obese rat	↓Loss of glomerular endothelial fenestrae, podocyte effacement, and slit pore diaphragm	Nistala et al. (2014) [69]
	↓Renal DPP-4 activity without changing renal DPP-4 protein levels	
	↓Proteinuria	
	↓Kidney tissue DPP-4 activity	
	↑Active GLP-1 in plasma	
	↑SDF-1α in kidney and plasma	
Wistar and Sprague–Dawley rat 5/6 nephrectomy	↓Interstitial fibrosis	Tsuprykov et al. (2016) [70]
	↓Glomerular hypertrophy	
	↓Albuminuria	
C57BL/6 mouse given peritoneal injection of free fatty acid-bound albumin	↓Tubular inflammation, fibrosis, and apoptosis	Tanaka et al. (2016) [71]
	↓Tubular injury	

Abbreviation: eNOS, endothelial nitric oxide synthase.

Renoprotective effects of linagliptin have also been seen in non-diabetic kidney disease. In a rat model of renal hypertension, linagliptin reduced oxidative stress but did not provide additional renoprotective effects [68]. However, in rats with obesity-related nephropathy, linagliptin reduced damage to the glomerular filtration barrier and proteinuria, while reducing DPP-4 activity in kidney tissue and increasing plasma levels of GLP-1 and SDF-1α [69]. Furthermore, in the 5/6 nephrectomy rat model of chronic kidney disease, linagliptin reduced albuminuria and kidney fibrosis [70]. In mice with 5/6 nephrectomy, the reductions in tubulointerstitial fibrosis and glomerulosclerosis resulting from linagliptin treatment occurred even in *Glp1r^−/−^* animals, indicating that these renoprotective effects were independent of GLP-1 signaling [72]. In another mouse model, linagliptin reduced the tubulointerstitial injury induced by peritoneal injection of free fatty acid-bound albumin, without altering blood glucose levels [71].

Other DPP-4 inhibitors have also demonstrated renoprotective effects in animal models of diabetic nephropathy, as reviewed elsewhere [18,19,73].

## Devil in the detail: putative molecular mechanisms of DPP-4-associated renoprotection

There is evidence to suggest that the pleiotropic renoprotective effects of linagliptin in animal models result from a number of different molecular mechanisms.

### Antifibrotic effects

Renal fibrosis, the final common pathway of progressive kidney diseases, disrupts kidney structure and thus reduces the organ’s filtration function [74–79]. The two main loci for renal fibrosis are the tubulointerstitial space and the glomerulus. As described above, linagliptin ameliorated kidney fibrosis (both tubulointerstitial fibrosis and glomerulosclerosis) and albuminuria in a murine model of type 1 diabetes without altering blood glucose levels [64]. This is consistent with studies showing antifibrotic effects of linagliptin in the heart [80–83], aorta [84] and peritoneum [85] in animal models. The antifibrotic changes in the kidneys occurred together with inhibition of the endothelial-to-mesenchymal transition (EndMT) [64], which is thought to be an important source of kidney fibroblasts [86–89] that play a key role in renal fibrosis [90]. Several different processes are responsible for the tissue accumulation of activated fibroblasts, which are the cells responsible for the establishment and progression of the fibrotic process via excessive production of collagen and other extracellular matrix proteins [91,92]. EndMT, the most recently discovered of these processes following its elucidation in 2007 [93], is a complex process in which cells detach from the endothelial layer, lose their specific molecular markers, and acquire a mesenchymal (more specifically, myofibroblastic) phenotype [91,92]. These myofibroblasts invade the interstitial space and express excessive quantities of proteins such as α-smooth muscle actin and type I collagen that are responsible for fibrosis. EndMT can be induced by several molecular pathways, the most important of which is initiated by transforming growth factor-β (TGF-β) [91,92].

In the above-described murine model of type 1 diabetes studied by the author and collaborators [64], immunohistochemical and Western blot analyses revealed that DPP-4 was up-regulated in the glomerular basement membrane, tubules, and peritubular vascular cells of the kidneys of diabetic mice compared with control mice; however, linagliptin reduced the expression and enzymatic activity of DPP-4 as well as the expression of TGF-β1 and TGF-β2 [64]. In a separate experiment in the same study, linagliptin inhibited TGF-β2-induced EndMT in cultured human dermal microvascular endothelial cells and reduced TGF-β2-induced phosphorylation of Smad3, a transcription factor that plays an essential role in TGF-β superfamily signaling [64]. Furthermore, analysis of microRNA (miR) profiles found that *miR-29a*, -*b*, and -*c* were suppressed in both diabetic kidneys and cultured endothelial cells but were restored by linagliptin treatment [64]. A binding site for *miR-29* was found in the 3′-UTR of DPP-4 and, using a reporter gene construct, *miR-29* was shown to suppress *DPP-4* gene expression [64]. These findings were confirmed in a separate study in a different animal model of chronic kidney disease, the 5/6 nephrectomy rat, where linagliptin treatment restored levels of *miR-29c* while suppressing the induction of pro-fibrotic miRs such as *miR-199-3p* [94].

**Figure 2:**
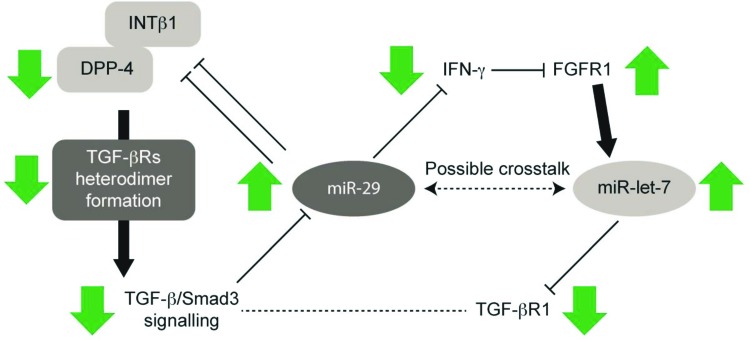
Potential anti-EndMT miR cross-talk between *miR-29* and *miR-let-7* DPP-4 inhibition suppresses the TGF-β signaling pathway, resulting in the induction of *miR-29. miR-29* could suppress DPP-4, integrin β1, and interferon-γ. Suppression of interferon-γ results in the induction of FGFR1; subsequently, *miR-let-7* is induced. Increased levels of *miR-let-7* are associated with suppression of the TGF-β receptor-1, resulting in much higher induction of *miR-29s*. Therefore, *miR-29* and *miR-let-7* comprise positive feedback loops of anti-EndMT programs. Abbreviations: IFN-γ, interferon-γ; INTβ1, integrin β1. Republished with permission of Wolters Kluwer from Takagaki et al. [19]. http://journals.lww.com/co-nephrolhypertens/Abstract/2017/01000/Dipeptidyl_peptidase_4_inhibition_and.11.aspx

Discovered approximately 20 years ago, miRs are a class of small (approximately 22 nts) non-coding RNA molecules that bind mRNA in the 3′-UTR to silence gene expression. A number of miRs are dysregulated in kidney fibrosis and other pathologies of diabetic nephropathy, including the *miR-29* family, which is generally suppressed during fibrosis of the kidney and other organs and is thus regarded as a signature miR of fibrotic diseases [95–97]. Target genes of *miR-29* include those encoding proteins of the extracellular matrix such as collagens, laminins, elastin, and integrin β1; therefore, *miR-29* in healthy tissues appears to suppress development of the extracellular matrix [96]. Also, *miR-29* targets the inflammatory cytokine interferon-γ, which participates in tissue fibrosis by suppressing expression of the fibroblast growth factor receptor 1 (FGFR1) and subsequently *miR-let-7* as well [97,98]. Profibrotic TGF-β/Smad signaling has been shown to down-regulate *miR-29* [96]. Similar to *miR-29*s, the *miR-let-7* family also has antifibrotic effects in the kidney of diabetic CD-1 mice [99], and there is bidirectional cross-talk between *miR-29*s and *miR-let-7*s in this model of diabetic nephropathy (Figure 2) [100].

Using the same STZ-induced CD-1 murine model of type 1 diabetes, the author and colleagues subsequently identified a new profibrotic molecular mechanism comprising an interaction between DPP-4 and integrin β1 in endothelial cells [65]. This interaction modulates TGF-β signaling to induce EndMT [65]. Integrins are transmembrane receptor proteins that play essential roles in cellular interactions with the extracellular matrix by binding to matrix proteins as well as other cell surface receptors [101]. Structurally, each integrin subunit comprises an extracellular domain involved in binding interactions, a transmembrane portion, and a short cytoplasmic tail that transduces extracellular–intracellular signals. The quaternary structure of integrins comprises 24 different αβ heterodimers formed from 18 α and 8 β subunits [101]. Integrin β1 has a diverse involvement in physiological and pathological processes, and plays a critical role in renal fibrosis by mediating TGF-β signaling [102–104]. Membrane-bound DPP-4 is critical for phosphorylation of the S785 residue of integrin β1, which plays a key role in binding to the extracellular matrix [105].

Crucially, the same recent study that identified the interaction between integrin β1 and DPP-4 that induces EndMT also showed that this interaction is disrupted by linagliptin [65]. In this study, levels of endothelial DPP-4, integrin β1, p-integrin β1, and TGF-β receptors were all higher in diabetic, fibrotic kidneys than in control murine kidneys. However, treatment with linagliptin reduced renal fibrosis and plasma cystatin C levels, and suppressed endothelial levels of DPP-4, integrin β1, p-integrin β1, and TGF-β receptors in diabetic kidneys. In cultured human dermal microvascular endothelial cells, there was a physical interaction between DPP-4 and integrin β1 that was increased in high glucose concentrations. Knockdown of DPP-4 by RNAi resulted in suppression of integrin β1 and the converse was also true. Furthermore, RNAi-mediated knockdown of either integrin β1 or DPP-4 also abolished TGF-β2-induced TGF-β receptor heterodimer formation, Smad3 phosphorylation, and EndMT. The interaction between DPP-4 and integrin β1 in endothelial cells also down-regulated expression of the VEGF receptor 2 (VEGF-R2) but up-regulated the expression of VEGF-R1 [65]. This would be expected to tip the balance of VEGF signaling toward EndMT, as VEGF-R1 stimulates EndMT while VEGF-R2 inhibits this fibrotic process [106,107].

**Figure 3:**
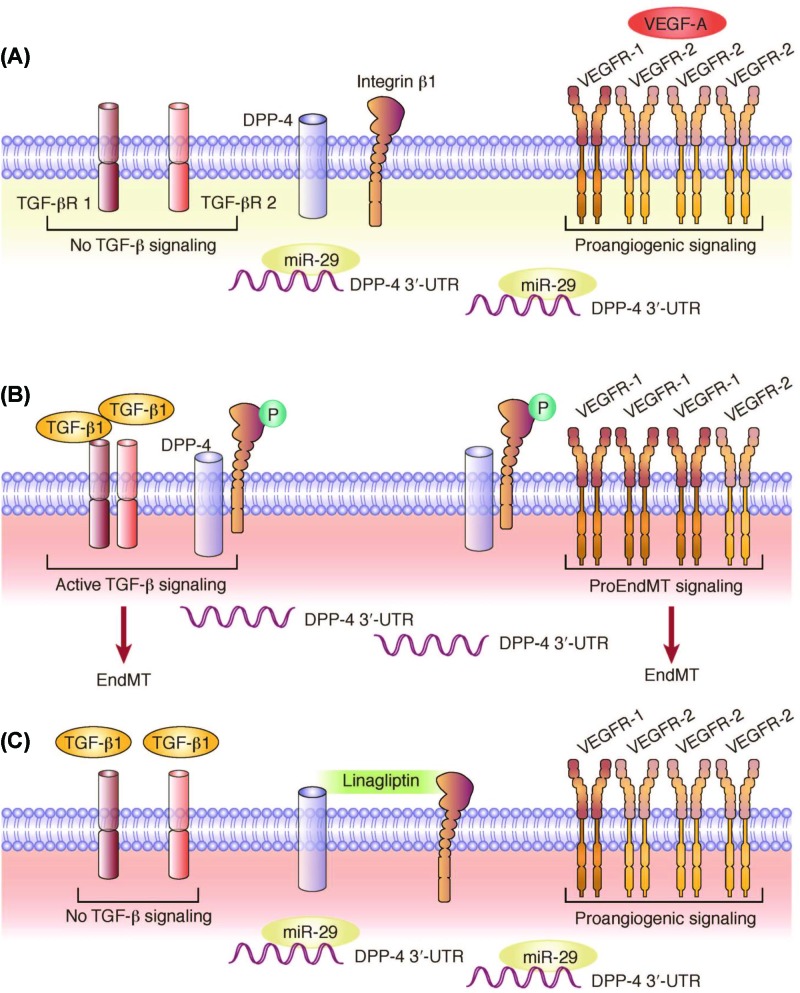
Schematic illustration of EndMT (**A**) Normoglycemia. Within endothelial cells in a normoglycemic microenvironment, *miR-29* keeps DPP-4 levels low. DPP-4 and integrin β1 do not interact. TGF-β receptors are not within active complexes. VEGF-R2 is more abundant than VEGF-R1, favoring proangiogenic VEGF signaling responses. (**B**) Hyperglycemia. Within a hyperglycemic microenvironment, *miR-29* is depleted. Accumulating DPP-4 interacts with integrin β1, resulting in its phosphorylation. DPP-4–integrin β1 complexes induce complex formation of TGF-β type I and type II receptors, enabling pro-EndMT signaling in response to TGF-β. Balance between VEGF receptors tilts toward VEGF-R1, favoring pro-EndMT signaling in response to VEGF-A. (**C**) Linagliptin effect in hyperglycemia. Linagliptin restores *miR-29* levels and inhibits DPP-4–integrin β1 interaction. Complex formation of TGF-β type I and type II receptors is impaired, blunting pro-EndMT signaling despite the presence of TGF-β ligands. VEGF-R2 is more abundant than VEGF-R1, favoring proangiogenic VEGF signaling responses. TGF-βR, transforming growth factor-β (TGF-β) receptor. Reprinted from Zeisberg and Zeisberg (2015) [110–112] with permission from the International Society of Nephrology.

Taken together, these studies [64,65] suggest the following pathophysiological role of DPP-4 in renal endothelial cells (Figure 3) [107,108]. The translational suppression of DPP-4 levels by *miR-29* binding of the 3′-UTR during normoglycemia is lost when *miR-29* levels decrease in hyperglycemic conditions. Subsequently, the newly abundant membrane-bound DPP-4 forms a complex with integrin β1 (which is also translationally suppressed by *miR-29* under normal conditions [109]), resulting in phosphorylation (activation) of the latter. This activated DPP-4–integrin β1 complex enhances heterodimer formation of TGF-β receptors and, consequently, TGF-β binding; in parallel, the DPP-4–integrin β1 complex stimulates VEGF-R1 expression – each of these effects leads to the profibrotic EndMT process [107]. Importantly, linagliptin inhibits these processes. Although it is not known if the interaction between membrane-bound DPP-4 and integrin β1 that results in phosphorylation of the latter is due to DPP-4 enzymatic activity or physical interaction, it is plausible that the antifibrotic effect observed in the recent study [65] via inhibition of the DPP-4–integrin β1 interaction is not a class effect but is specific for linagliptin, as noted by the accompanying commentary [107]. The reasons why the antifibrotic renoprotective properties of linagliptin may not be shared by other DPP-4 inhibitors are discussed later in this article.

Linagliptin has also been shown to reduce TGF-β signaling in human kidney proximal tubular epithelial (HK-2) cells in high glucose concentrations by a different mechanism [110–112]. The cation-independent mannose 6-phosphate receptor (CIM6PR) activated TGF-β1 in HK-2 cells exposed to high glucose [110]; under the same conditions, linagliptin inhibited TGF-β1 activation in this cell line [111], an effect that was due to disruption of the protein–protein interaction between DPP-4 and CIM6PR [112]. It is unclear how linagliptin disrupts the physical interaction between DPP-4 and CIM6PR but, given the differences in chemical structure amongst DPP-4 inhibitors, again, it is possible, even plausible, that this is not a class effect.

In light of these findings, it is notable that linagliptin treatment significantly reduced serum levels of TGF-β1 in a double-blind, randomized, placebo-controlled, mechanistic study in non-diabetic, hypertensive patients [113].

### Other putative renoprotective molecular mechanisms of linagliptin

In addition to its antifibrotic properties described above, linagliptin may protect the kidneys via several other pathways, including effects on AGE-RAGE signaling, oxidative stress, inflammation, endothelial nitric oxide activity, and increased levels of the DPP-4 substrates SDF-1 and GLP-1. The studies underpinning these hypotheses are discussed as follows.

### AGE-RAGE pathway, oxidative stress, endothelial nitric oxide activity

Diabetes is associated with increased levels of AGEs, i.e. proteins or lipids that are glycated by the non-enzymatic Maillard reaction as a consequence of their exposure to glucose and other saccharides. AGEs contribute to development of the macro- and microvascular complications of diabetes, including chronic kidney disease − both by cross-linking to molecules in the extracellular matrix basement membrane and by binding the RAGE receptor [114]. Activation of RAGE by AGEs triggers oxidative stress, inflammation, and other pathogenic processes [114]. In cultured human umbilical vein endothelial cells, soluble DPP-4 was shown to increase oxidative stress and expression of RAGE, seemingly via binding of CIM6PR; both effects were blocked by linagliptin, which also inhibited AGE-induced increases in DPP-4 levels [115]. As described above, linagliptin also blocked AGE-RAGE signaling in a rat model of type 1 diabetes [62]. On the basis of these and other studies, it appears that there is a cross-talk between AGE-RAGE signaling and the DPP-4/incretin system, which may represent a novel therapeutic target for preventing vascular complications of type 2 diabetes [116].

Linagliptin has antioxidant properties not shared by other DPP-4 inhibitors [117]. This may reflect its unique chemical structure within the DPP-4 inhibitor class of drugs: linagliptin alone contains a xanthine-based scaffold and can inhibit xanthine oxidase [118], an enzyme of purine metabolism that generates reactive oxygen species. Reduced renal oxidative stress was associated with linagliptin treatment in several of the animal models described above [61,62,66,68]. Furthermore, in a murine model of diabetes, linagliptin treatment reduced albuminuria and renal hypertrophy in a glucose-independent manner, but only in mice with wild-type antioxidant function [119]. In these mice, linagliptin also increased levels of the antioxidant enzymes catalase and manganese superoxide dismutase; however, in mice with reduced antioxidant function resulting from knockout of glucose 6-phosphate dehydrogenase, linagliptin neither increased these antioxidant enzymes nor decreased albuminuria or kidney hypertrophy. These findings suggest that the renoprotective effects of linagliptin in this model were elicited mainly via its antioxidant properties [119]. The antioxidant effects of linagliptin might also block positive feedback between the generation of reactive oxygen species and AGE-RAGE signaling in diabetic nephropathy.

The endothelium plays an important role in maintaining vascular homeostasis. Impairment of nitric oxide-mediated vasodilation (endothelial dysfunction) is closely associated with the development of diabetic nephropathy. In an *in vivo* model of septic shock, linagliptin ameliorated vascular dysfunction as well as reducing oxidative stress and inflammation [117]. Furthermore, *in vitro*, linagliptin was not only the most potent of the gliptins tested at inhibiting both the oxidative burst by isolated activated human neutrophils and their adhesion to endothelial cells, it was also the most potent direct vasodilator of isolated aortic rings [117]. In a subsequent study comprising both *ex vivo* and *in vitro* experiments, linagliptin was shown to directly interact with the caveolin-1/endothelial nitric oxide synthase (eNOS) complex to rescue eNOS activity; this effect was independent of both glucose-lowering and GLP-1 receptor signaling [120]. Furthermore, linagliptin − but not sitagliptin or vildagliptin – ameliorated endothelial dysfunction induced by high glucose concentrations *in vitro* [121].

### Non-GLP-1 substrates of DPP-4

SDF-1 is a chemokine that promotes endothelial repair by mobilizing endothelial progenitor cells from bone marrow, and it has been shown to mediate repair of cells and tissues during ischemic kidney injury [122,123]. As described earlier, SDF-1 is a physiological substrate of DPP-4 [32]. In the previously described study in *Glp1r^−/−^* Akita diabetic mice, linagliptin up-regulated SDF-1 expression in distal tubules of the kidney together with ameliorating kidney pathology and reducing renal oxidative stress [66]. Linagliptin also increased renal SDF-1 expression and plasma SDF-1 levels in rats with obesity-related nephropathy, with concomitant reductions in renal DPP-4 activity, damage to the glomerular filtration barrier, and proteinuria [69]. Of related interest, linagliptin reduced infarct size in a rat model of cardiac ischemia/reperfusion injury, an effect that was associated with a significantly increased number of cells positive for SDF-1α and its receptor (CXCR4) near to and within the infarcted area [124]. Importantly, linagliptin was shown to elevate plasma SDF-1 in type 2 diabetes patients with or without chronic kidney disease in a randomized, crossover, placebo-controlled trial [125]. SDF-1 elevating effects have also been seen in small clinical studies of sitagliptin [126,127].

### GLP-1

The GLP-1 receptor is expressed in many non-pancreatic tissues, including the kidney. However, its exact localization within this organ has not yet been robustly characterized, due to insufficient sensitivity and specificity of commercially available antisera [128]. The physiological role of GLP-1 within the kidney is also incompletely understood but appears to encompass natriuretic effects mediated by inhibition of NHE3 in the proximal tubule [129]. Interestingly, DPP-4 appears to modulate NHE3 activity in a GLP-1-independent manner [43]. The renal effects of GLP-1 and GLP-1 receptor agonists are reviewed elsewhere [128,130], and their detailed description is beyond the scope of this review. As described above, many of the putative renoprotective effects of linagliptin have been seen in *Glp1r^−/−^* animals, indicating that GLP-1 receptor signaling alone is unlikely to account for all of the renoprotective effects of this DPP-4 inhibitor.

## Renoprotective class effect of DPP-4 inhibitors? Not so fast

All licensed DPP-4 inhibitors are orally administered small molecules that inhibit plasma DPP-4 activity by >80%, which consequently raises the plasma concentration of GLP-1 by two to three fold [20]. Elevating GLP-1, and thus its insulinotropic effect, is thought to be the primary mechanism by which DPP-4 inhibitors elicit their glucose-lowering effects, and members of this drug class appear to be approximately equivalent in terms of their antihyperglycemic clinical efficacy.

However, despite sharing a common mechanism of action, DPP-4 inhibitors comprise a chemically heterogeneous class of molecules with important differences in pharmacokinetics (Table 2) [20]. Notably, linagliptin is the only one of the five globally marketed DPP-4 inhibitors to be excreted from the body mainly by non-renal pathways, a consequence of its high level of protein binding and thus low concentration of free drug. In contrast, others (sitagliptin, vildagliptin, saxagliptin, and alogliptin) are predominantly removed via the kidneys, which necessitates their dose reduction in patients with kidney disease [24–27]. This was demonstrated in a head-to-head preclinical study comparing linagliptin, sitagliptin, and alogliptin in a rat model of chronic kidney disease. In this study, linagliptin was the only DPP-4 inhibitor whose exposure was not increased by renal impairment, and which did not elevate markers of tubular and glomerular injury [131]. Furthermore, linagliptin normalized the expression of the following key molecular markers of uremic cardiomyopathy in this model: TGF-β, tissue inhibitor of matrix metalloproteinase-1 (TIMP-1), and procollagen type 3 α1 (Col3α1) [131]. Similarly, in a separate study utilizing a rat model of uremic cardiomyopathy, linagliptin prevented the development of cardiac diastolic dysfunction without affecting renal function [132]. Theoretically, other DPP-4 inhibitors that are excreted in the urine may modify the proteolytic activity of apical membrane-bound DPP-4 in the proximal tubule [133].

**Table 2:** Key pharmacological properties of linagliptin and other globally marketed DPP-4 inhibitors

Compound	IC_50_ (nM) [134]	*K*_D_ (nM) [134]	*k*_on_ (M^−1^.s^−1^) [134]	*k*_off_ × 10^−4^ (s^−1^) [134]	*V*_D_ [20]	Renal excretion (%) [20]	Protein binding (%) [20]
Alogliptin	35.5	2.4	1.3 × 10^6^	31	581	63.3	20
**Linagliptin**	**1.4**	**0.0066**	**7.6 × 10^6^**	**0.51**	**1110−3060**	**6.3**	**75−99**
Saxagliptin	55.0	0.3	9.2 × 10^5^	2.0	151	24	<10
Sitagliptin	45.0	5.3	1.5 × 10^7^	630	198	75	38
Vildagliptin	95.5	2.4	7.1 × 10^4^	1.7	70.5	22.6	9

Abbreviations: *K*_D_, equilibrium dissociation constant; *k*_on_, rate constant for association of the DPP-4/inhibitor complex; *k*_off_, rate constant for dissociation of the DPP-4/inhibitor complex; *V*_D_, volume of distribution.

Another intraclass difference in pharmacokinetics with consequences for renoprotection is the large volume of distribution of linagliptin compared with other DPP-4 inhibitors, indicating greater tissue penetration with the former. In fact, the ability of linagliptin to penetrate deep into kidney tissue has been demonstrated [135,136]. In an *in vivo* study of the tissue distribution of linagliptin in wild-type and DPP-4-deficient rats using whole-body autoradiography and measurement of tissue radioactivity following administration of radiolabeled compound, the highest drug concentrations were located in the kidneys and liver [135]. A follow-up study employing high-resolution autoradiography found that linagliptin in the kidney was located mainly on glomerular podocytes and on the brush border microvilli of the proximal tubules, with a similar distribution pattern to that of DPP-4 itself [136]. These data suggest that linagliptin is able to reach all DPP-4-containing compartments of the kidney.

*In vitro* potency and enzyme-binding kinetics also differ between DPP-4 inhibitors (Table 2) [134]. With an IC_50_ of approximately 1 nmol/l, linagliptin is the most potent DPP-4 inhibitor [134,137]. Additionally, a comparative analysis of binding kinetics of the five globally marketed DPP-4 inhibitors found that linagliptin had the highest binding affinity for DPP-4 (*K*_D_ = 0.0066 nmol/l), one of the fastest binding rates (*k*_on_ = 7.6 × 10^6^ M^−1^.s^−1^), and the slowest dissociation rate (*k*_off_ = 5.1 × 10^−5^ s^−1^) [134,138].

These pharmacological differences suggest that other DPP-4 inhibitors may not necessarily have the same *in vivo* pleiotropic effects as linagliptin. Several studies have explored this possibility. In an *ex vivo* study in Zucker diabetic fatty (ZDF) rats, linagliptin conferred greater vascular protection than sitagliptin despite similar effects on blood glucose levels [139]. In this study, ZDF rats were administered linagliptin, sitagliptin, or placebo for 4 weeks. After both the first and last treatments, reductions in blood glucose and plasma DPP-4 activity, and increases in plasma insulin, were equivalent with linagliptin and sitagliptin. However, acetylcholine-induced vascular relaxation in isolated arteries was greater with linagliptin than sitagliptin, as was inhibition of membrane-bound DPP-4 activity, while lipid peroxidation was lower [139]. In an *in vitro* study, linagliptin but not sitagliptin suppressed DPP-4 enzymatic activity and protein levels in TGF-β2-treated cultured human dermal microvascular endothelial cells [140]. Furthermore, linagliptin but not sitagliptin inhibited EndMT, restored TGF-β2-induced changes in *miR-29a–c* and VEGFR levels, suppressed TGF-β2-induced increases in integrin β1 levels, and decreased DPP-4 dimerization [140]. In a comparative *ex vivo* study, linagliptin elicited more sustained inhibition of *in situ* DPP-4 activity than sitagliptin in the proximal tubule and glomerulus of normal male Wistar rats administered single oral doses of these drugs [138]. Furthermore, linagliptin but not sitagliptin or vildagliptin inhibited renal DPP-4 activity in a rat model of renal ischemia–reperfusion injury, albeit this did not correlate with amelioration of histopathologically assessed tubular damage [141].

## Elephant in the room: clinical kidney protection with DPP-4 inhibitors?

Despite the many studies described here showing renoprotective effects of linagliptin and other DPP-4 inhibitors in animal models of diabetic nephropathy and non-diabetic kidney disease, as yet there is little clinical data to support the hypothesis that these drugs have pleiotropic renal benefits. Although this may simply reflect the paucity of clinical studies designed specifically to evaluate renal outcomes with DPP-4 inhibitors, some relevant findings have been reported.

A *post hoc* analysis of the SAVOR-TIMI 53 cardiovascular safety study found clinically meaningful reductions in albuminuria with saxagliptin treatment but no concomitant improvements in either eGFR or hard renal outcomes such as initiation of dialysis or renal transplant. The albuminuria-lowering effect of saxagliptin was not associated with its effect on glycemic control [142]. In the similar TECOS study of sitagliptin, there were no clinically meaningful changes in albuminuria [143]. While these studies were not designed to investigate renoprotective effects, neither was the LEADER cardiovascular safety study in which the GLP-1 receptor agonist liraglutide did appear to improve renal outcomes, as measured using a composite end point comprising new-onset persistent macroalbuminuria, persistent doubling of serum creatinine level, end-stage renal disease, or death due to kidney disease (hazard ratio: 0.78; 95% CI: 0.67–0.92) [144]. However, the reduced risk for adverse renal outcomes measured by this composite end point was driven mainly by the ‘ soft’ outcome of reduced incidence of macroalbuminuria. These different effects of a GLP-1 receptor agonist compared with two DPP-4 inhibitors would be consistent with the renal effects of all three drugs being exerted via GLP-1, as liraglutide elicits pharmacological levels of GLP-1 signaling while saxagliptin and sitagliptin elevate GLP-1 only to high physiological levels. In a 12-week, randomized, double-blind, placebo-controlled clinical trial in 55 insulin-naïve patients with type 2 diabetes, treatment with sitagliptin did not affect renal hemodynamics [145]. Interestingly, however, a prospective cohort study found that treatment with DPP-4 inhibitors was associated with a reduced risk of acute kidney injury in patients with diabetes [146].

As have other DPP-4 inhibitors, linagliptin has demonstrated glucose-lowering efficacy and tolerability in type 2 diabetes patients with kidney disease [147,148]. Linagliptin has also demonstrated efficacy and tolerability in type 2 diabetes patients with hypertension and microalbuminuria [149]. Interestingly, a pooled analysis of four 24-week, randomized, placebo-controlled clinical trials designed to evaluate glycemic efficacy found that linagliptin treatment was associated with a significant 32% reduction in urinary albumin-to-creatinine ratio (UACR) in individuals with albuminuria (UACR: 30−3000 mg/g) who were already receiving standard of care for diabetic nephropathy (angiotensin-receptor blockers or angiotensin-converting enzyme inhibitors) [150]. Furthermore, a pooled analysis of 13 randomized, placebo-controlled clinical trials revealed a significant mean reduction in adverse renal events in patients receiving linagliptin [151]. However, as the underlying studies were not designed to evaluate renal outcomes, these findings are hypothesis generating only. In fact, in the subsequent 24-week, MARLINA-T2D™ clinical trial, which was designed to investigate potential albuminuria-lowering effects in patients with early type 2 diabetes, linagliptin elicited only a non-significant 6% reduction in UACR [152]. Nevertheless, as has been described here, renoprotective effects of this DPP-4 inhibitor may be predominantly mediated by its antifibrotic actions, which would not necessarily manifest as changes in albuminuria over the short-term in patients with early type 2 diabetes.

A mechanistic, parallel-group, randomized clinical study in 62 patients with early type 2 diabetes suggested that 4 weeks of treatment with linagliptin prevented impairment of renal endothelial function, as measured by changes in basal renal endothelial nitric oxide activity [153]. The glomerular hyperfiltration that characterizes early diabetic nephropathy is associated with increased basal nitric oxide activity [154,155]. Another mechanistic, randomized clinical study found that 4 weeks of linagliptin treatment improved microvascular endothelial function compared with both placebo and the sulphonylurea, glimepiride [156]. In this crossover study in 42 patients with early type 2 diabetes, there was no significant change in macrovascular endothelial function measured by brachial flow-mediated vasodilation. However, there were significant improvements in fasting microvascular function, as measured by changes in blood flow on the dorsal thenar site of the right hand recorded with laser-Doppler flowmetry [156].

## Conclusion

The renoprotective effects of improving glycemic control are well established, and linagliptin and other DPP-4 inhibitors are therefore anticipated to provide such benefits via their glucose-lowering properties alone. Additionally, linagliptin has demonstrated pleiotropic renoprotective properties in diabetic and non-diabetic animal models of nephropathy – notably, antifibrotic effects mediated via interaction with miR and integrins. The antioxidant properties of linagliptin also seem likely to play a potentially unique renoprotective role. Additional effects of linagliptin in disrupting AGE-RAGE signaling, increasing levels of peptides such as GLP-1 and SDF-1, ameliorating endothelial dysfunction, and reducing inflammation are also likely to be important for renoprotection. Looking to the future, technologies such as metabolomics [157] and peptidomics [70] that can simultaneously evaluate all biomolecules of a particular type seem well suited to identifying molecular pathways leading to renoprotection with DPP-4 inhibitors – given the large number of biopeptides with potential cardiorenal effects whose physiological levels could be affected by inhibition of DPP-4. Peptidomics has already demonstrated the ability to identify global changes in peptide levels in the kidney and plasma resulting from linagliptin treatment [70].

Despite a common mechanism of action, DPP-4 inhibitors comprise a heterogeneous class of molecules with clinically relevant differences in pharmacology. Unlike other members of this drug class, linagliptin is non-renally excreted but capable of penetrating the various compartments of the kidney. Thus, based on its pharmacology and animal studies to date, linagliptin appears to offer the greatest potential for renoprotection.

As with other incretin therapies, the putative renoprotective properties of linagliptin have yet to be demonstrated convincingly in clinical trials; however, this may simply reflect the scarcity of studies designed to specifically measure renal outcomes. For this reason, there is much interest in the ongoing CARMELINA® study. This large event-driven, placebo-controlled clinical trial is the first study designed and adequately powered to robustly evaluate renal outcomes of treatment with a DPP-4 inhibitor. Almost 7000 type 2 diabetes patients with high cardiorenal risk have been enrolled in CARMELINA® [158] and results are anticipated in 2018.

## Abbreviations

AGE: advanced glycation end product
CARMELINA®: Cardiovascular and Renal Microvascular Outcome Study with Linagliptin in Patients with Type 2 Diabetes Mellitus
CD: cluster of differentiation
CIM6PR: cation-independent mannose 6-phosphate receptor
CXCR4: C-X-C chemokine receptor type 4
DPP-4: dipeptidyl peptidase-4
eGFR: estimated glomerular filtration rate
EndMT: endothelial-to-mesenchymal transition
eNOS: endothelial nitric oxide synthase
GLP-1: glucagon-like peptide-1
HK-2: human kidney proximal tubular epithelial
NHE3: sodium-hydrogen exchanger-3
RAGE: receptor for AGE
SDF-1: stromal cell derived factor-1
SGLT2: sodium-glucose cotransporter-2
STZ: streptozotocin
TGF-β: transforming growth factor-β
UACR: urinary albumin-to-creatinine ratio
VEGF: vascular endothelial growth factor
VEGF-R: VEGF receptor
