# Correction: The role of renal dipeptidyl peptidase-4 in kidney disease: renal effects of dipeptidyl peptidase-4 inhibitors with a focus on linagliptin

**DOI:** 10.1042/CS20180031_COR

**Published:** 2019-01-15

**Authors:** Keizo Kanasaki

**Clinical Science (2018) 132(4), 489–507; https://doi.org/10.1042/CS20180031**

The published article contains several errors introduced during the production process.

1. In the penultimate sentence of the Introduction, the web address, ClinicalTrials.gov incorrectly linked to a reference. The correct sentence is presented here:
  Furthermore, linagliptin is the first and so far only DPP-4 inhibitor to be evaluated in a randomized clinical trial designed to robustly assess renal outcomes: the ongoing Cardiovascular and Renal Microvascular Outcome Study with Linagliptin in Patients with Type 2 Diabetes Mellitus (CARMELINA®; ClinicalTrials.gov: NCT01897532).
2. In the captions to Figures 1 and 3, the reference citations attributed to statements of permission to reproduce were incorrect; the correct sources of the figures are presented here.

- Figure 1 was reproduced from reference [18]: Shi, S., Koya, D. and Kanasaki, K. (2016) Dipeptidyl peptidase-4 and kidney fibrosis in diabetes. Fibrogenesis Tissue Repair **9**, 1, https://doi.org/10.1186/s13069-016-0038-0
- Figure 3 was reproduced from reference [107]: Zeisberg, M. and Zeisberg, E.M. (2015) Evidence for antifibrotic incretin-independent effects of the DPP-4 inhibitor linagliptin. Kidney Int. **88**, 429–431, https://doi.org/10.1038/ki.2015.175
  Corrected online 8 January 2019.

